# The COVID-19 Pandemic and the Resilience of the Pharmaceutical Supply Chain: Lessons from Past Experiences and Strategies for the Future

**DOI:** 10.5812/ijpr-152723

**Published:** 2024-11-05

**Authors:** Mohamad Ali Aivazi, Hamid Reza Rasekh, Mohammad Peikanpour, Farzad Peiravian, Sajjad Esmaeili, Leila Zarei

**Affiliations:** 1 Department of Pharmacoeconomics and Pharma Management, School of Pharmacy, Shahid Beheshti University of Medical Sciences, Tehran, Iran; 2 Health Policy Research Center, Institute of Health, Shiraz University of Medical Sciences, Shiraz, Iran

**Keywords:** COVID-19, Pharmaceutical Supply Chain, Resilience, Vulnerabilities, Capabilities

## Abstract

**Background:**

The pharmaceutical supply chain (PSC) faced numerous challenges, particularly during the COVID-19 crisis. Due to the supply chain (SC) 's vulnerabilities, it requires enhanced capabilities to address these challenges. In Iran, specific economic and political issues have intensified the vulnerabilities of the PSC.

**Objectives:**

This study investigates the issues caused by the COVID-19 crisis in the PSC, identifies and characterizes these issues, and recommends appropriate courses of action to address future SC disruptions.

**Methods:**

This study is a qualitative-quantitative analysis conducted in Iran during the COVID-19 crisis. Qualitative thematic analysis was performed from July 2022 until May 2023. Semi-structured, in-depth, face-to-face interviews with 23 Iranian PSC specialists were conducted until saturation was reached. The qualitative phase was analyzed using MAXQDA 2021. The quantitative phase included a survey of 547 individuals working in pharmaceutical manufacturing in Iran, with the questionnaires analyzed using SPSS 26.

**Results:**

In the qualitative phase, the research identified two main themes: (1) vulnerabilities and (2) capabilities, along with 15 subthemes providing solutions to enhance the resilience of the PSC. In the quantitative phase, findings from 64 questionnaires highlighted major vulnerabilities and capabilities necessary to create a resilient SC. The median score for vulnerabilities was 5.12, while the median score for capabilities was 5.39.

**Conclusions:**

According to the questionnaire results, the quantitative findings indicate that capabilities received a higher score, suggesting that this sector of the PSC demonstrated better resilience against the pandemic. This study, with its contextual focus, mixed-method approach, comprehensive analysis of vulnerabilities and capabilities, and sector-specific insights, offers a novel contribution to the understanding of SC resilience within Iranian pharmaceutical manufacturing. It also has the potential to promote further research in other sectors of the PSC.

## 1. Background

Today’s competition is no longer between companies but between their respective supply chains (SCs) (1). A SC refers to a complex system of interconnected organizations responsible for delivering products, services, money, and information from suppliers to customers (2). 

The pharmaceutical supply chain (PSC) manages the transformation of raw materials into finished products and their distribution to end buyers, ensuring the timely delivery of essential drugs in compliance with standards (3). The PSC must be highly flexible due to the health and safety risks posed by breakdowns or defects and requires continuous tracking systems to maintain the uninterrupted flow of essential goods (4).

Every process and decision within an organization is subject to uncertainty in the business environment. While PRO errors can lead to unexpected events due to incorrect computations and judgments, these uncertainties require continuous monitoring and management. Failure to recognize such events in time can have significant consequences (5). No system, including the PSC, is completely secure worldwide (4).

The pharmaceutical sector faces various challenges, particularly in marketing, research and development (R&D), pharmaceutical lifecycle management, government regulations, patent durations, production costs, and SC management. Among these complexities, the resilience of the SC holds particular importance (4).

Resilience is defined as the inherent capacity, capability, and skill of a system to endure, evolve, and thrive while confronting disruptive changes, or as the ability of the system to survive and adapt to new situations. Resilient systems can grow and improve even in chaotic conditions (6).

Resilience in SC management is a crucial skill that involves managing risks, opening multiple distribution paths for suppliers, and fostering strong relationships with stakeholders (7). Supply chain resilience (SCR) refers to the SC's ability to absorb changes efficiently with minimal disruption while continuing operations amidst uncertainties and unforeseen challenges. It implies the ability to recover from disturbances and carry out routine operations with little harm (8). The strength and durability of a company’s operations are closely linked to the resilience of its SC. Since a company relies on its suppliers for uninterrupted processes and on its customers for sustained revenue, the robustness of the entire SC directly influences its overall resilience (9).

The concept of SCR has gained more attention due to the growing challenges in SCs, such as global events, disasters, and emergencies (10). 

Several novel contagious diseases over the last thirty years have severely impacted public health and safety systems. By incorporating resilience into health systems, the capacity to adjust and respond to emerging health hazards is significantly enhanced, thus reinforcing the system’s ability to sustain operations and effectively counter new health threats (11).

During COVID-19, businesses around the world were disrupted, most notably in the global SC. Over 94% of Fortune 1000 companies reported SC disruptions caused by the pandemic. Nearly all companies, particularly multinational corporations (MNCs), faced problems related to SC disruptions. The pandemic affected supply, demand, and logistics across the chain. Production shutdowns in countries that were key hubs in global production networks led to shortages of essential items, from hand sanitizers to surgical masks (12). The sudden lockdowns, border closures, and restrictions on movement caused numerous logistical issues (13, 14). Many MNCs attempted to modify their SC strategies, shifting from global to domestic, allowing local suppliers to integrate into the global value chain (15, 16).

Scholars agree that SCs need to be more flexible to cope with the volatility and variety of disruptions (17, 18). Such SCs would be more responsive, reducing uncertainty and risk, and addressing the wide range of disruptive effects that occurred as a result of the COVID-19 pandemic. Stock policies should be adjusted to the ever-changing business conditions that developed during the pandemic (13, 15). It is essential to conduct a detailed analysis of COVID-19 pandemic-related disruptions across the SC and their impacts. We must recognize the complexity of the situation and the confusion caused by the pandemic. Furthermore, a comprehensive view of the disruptions caused by the COVID-19 pandemic and their implications on the entire SC environment is required (19).

The COVID-19 pandemic had a substantial impact on the pharmaceutical sector in low-income countries, particularly by limiting access to high-quality and affordable medications (20, 21) and negatively affecting the socioeconomic status and livelihoods of people in these societies (22). Improving medication accessibility in areas with limited resources became imperative (20, 21).

A resilient pharmaceutical industry can provide accessible, high-quality healthcare services, especially during pandemics. As highlighted by recent events, the PSC may experience severe disruptions for various reasons. For example, many barriers may prevent the development and advancement of innovative technologies, including overreliance on traditional manufacturing and distribution channels, such as those in China and India, which results in vulnerabilities in the global SC (23, 24). During pandemics, the PSC must administer advanced SC management systems to resist disruption and ensure a steady flow of high-quality pharmaceutical products. It is also suggested that the pharmaceutical industry diversify its manufacturing and distribution routes. This ensures that when certain routes are temporarily or completely unavailable, firms are not severely impacted (24).

COVID-19 was the most notable crisis in recent years. Considering the significant impact of the pandemic on the PSC and the necessity of mitigating risks posed by other unpredictable events in the future, resilience is the most critical factor. 

## 2. Objectives 

Consequently, in this study, we aimed to assess the resilience of the PSC in Iran during the COVID-19 pandemic. We also sought to draw lessons from past experiences and develop strategies for the future.

## 3. Methods

This mixed-method research was conducted in 2022 - 2023 in Iran. The quantitative phase involved a survey using a Self-Administered Questionnaire, derived from the qualitative phase, and completed by 547 eligible participants. To eliminate bias, a purposive sampling method was employed in the qualitative phase, and the questionnaire was designed and approved by PSC experts.

### 3.1. Qualitative Phase

We initiated semi-structured interviews with experts based on Pettit et al.'s resilience framework (8). A total of 23 face-to-face interviews were conducted with participants who met the inclusion criteria, from July 2022 to May 2023 in Tehran, Iran. The inclusion criteria were purposive sampling. Participants in the study included health policymakers, insurance experts, raw material suppliers, pharmaceutical company managers, distribution company managers, and pharmacists from both public and private pharmacies, each with a minimum of five years of practice and a well-established national reputation as an authority in the relevant field. To ensure a wide range of perspectives, purposive and snowball sampling techniques were used. Sampling continued until data saturation was achieved.

#### 3.1.1. Concept of Resilience in the Supply Chain

(1) Vulnerabilities: Factors that predispose an organization to disruption. 

(2) Capabilities: Characteristics that empower an enterprise to navigate and overcome disruptions, thereby sustaining the functionality of its SC (8).

The interviews identified factors related to vulnerabilities and capabilities based on Iran's unique conditions. 

The investigation and dissemination of this study were conducted using the Standards for reporting qualitative research (SRQR) (25) and the consolidated criteria for reporting qualitative research (COREQ) (26) as guiding instruments.

In-depth, semi-structured interviews were initially conducted by the first author, M.A.A., in a face-to-face setting, using a pre-tested interview guide. This guide consisted of 14 questions focused on the resilience of the PSC in Iran during the COVID-19 crisis. Each interview lasted an average of 75 minutes.

Data collection occurred concurrently with analysis, following the thematic analysis approach. Goldsmith's five-step framework analysis method was applied to the data analysis (27). Guba and Lincoln's four standards were used to determine the validity of the data (28). The software utilized for data analysis was MAXQDA 2020.

### 3.2. Quantitative Phase

A cross-sectional survey was conducted in 32 top pharmaceutical manufacturing companies by market share to collect data in this phase, using a Self-Administered Questionnaire from June 2023 to December 2023 in Iran. The final questionnaire consisted of 64 questions. According to Pettit et al. (8) and Ekanayake et al. (29), the two main components of the questionnaire were vulnerability and capability (with 32 questions divided into seven and eight categories, respectively). The questionnaires were distributed to senior managers, technical managers, and personnel from the marketing, commerce, technical/engineering, finance, R&D, production, quality assurance, and quality control departments. Data were measured using the 7-point Thurstone Scale. Some questions also gathered respondents’ demographic information (Appendix 1 in Supplementary File).

Before conducting the survey, a pilot study was conducted. Cronbach's alpha indicated an acceptable level of internal consistency (Appendix 2 in Supplementary File). Ten eligible individuals assessed face and content validity, providing feedback on elements to be included or excluded from the questionnaire. All participants ultimately agreed upon the questionnaire's suitability (4).

To determine the required number of participants in the quantitative phase, Cochran's formula was applied, indicating a minimum of 385 respondents. Thus, we distributed questionnaires to 960 individuals, and 547 responses were received, yielding a 57% response rate. The data were analyzed using SPSS software (version 25).

## 4. Results

### 4.1. Qualitative Phase 

The interviewees primarily consisted of CEOs of manufacturing and distribution companies, officials from the Food and Drug Organization, the Vice President of Food and Drug at one of the Universities of Medical Sciences, board members of the Syndicate of Human Drug Industry Owners and the Syndicate of Pharmaceutical Active Ingredients Manufacturers, managers of insurance companies, members of the Iranian Pharmacists Association, and managers of public and private pharmacies. The respondents ranged in age from 39 to 69 years. All participants were male, with most holding a degree in pharmacy, and a few possessing a PhD in pharmacy.

The findings identified vulnerabilities and capabilities as the main themes, which included 15 subthemes (seven vulnerabilities and eight capabilities) related to the resilience of the PSC during the COVID-19 crisis. Additionally, various codes were identified to represent coping strategies aimed at enhancing resilience within the PSC. Examples of interviewees' statements can be found in Appendix 3 in Supplementary File.

#### 4.1.1. Vulnerabilities

##### 4.1.1.1. Organizational 

This refers to the organization's inability to establish protocols and effectively manage its workforce.

##### 4.1.1.2. Information Technology

This vulnerability is related to limited and slow access to information, as well as an inadequate IT infrastructure.

##### 4.1.1.3. Procedural

This refers to shortcomings in planning, implementation, and achieving results, as well as disruptions in transportation. Several PSC members highlighted gaps in planning, with some having worked in senior positions for over twenty years. It's important to note that discussions about how goods are delivered are frequently directed at the pharmaceutical industry in Iran and other parts of the world.

##### 4.1.1.4. Financial

This category includes extreme events likely to cause financial (FIN) distress to businesses, such as currency fluctuations and lack of liquidity. A notable point during the pandemic was that distributors primarily sought to buy drugs from manufacturers of COVID-19 therapeutic agents.

##### 4.1.1.5. Control/Supportive

This category relates to the insufficiency in oversight and support provided by policy-making bodies. Unfortunately, for various reasons, there were issues with the surveillance of commodities.

##### 4.1.1.6. Supplier/Customer

This category addresses vulnerabilities related to suppliers and customers, including factors such as a lack of trust between suppliers or customers, involvement of inadequately trained staff, product or contract fraud, and supply-demand mismatches.

##### 4.1.1.7. Environmental/External

Frequent changes in external factors beyond the firm’s control can have indirect effects, imposing restrictions or constraints on the organization’s operations (8). 

The welfare and health of the target population may be compromised by economic constraints. In Iran’s case, the literature review findings indicated that sanctions worsened the population’s situation by negatively impacting the affordability, availability, and quality of medical care.

#### 4.1.2. Capabilities 

##### 4.1.2.1. Flexibility 

Flexibility (FLX) refers to the ability to mobilize resources quickly when needed. This FLX can be demonstrated in both the order fulfillment process and the sourcing process (8). It allows for resource replacement, the application of innovations, the creation of systems to manage temporary changes, and the ability to handle different orders.

##### 4.1.2.2. Agility

The ability to promptly adjust to changes, either by restoring the initial condition or by rearranging the system (30, 31). Agility (AGL)cannot be limited to a single firm; it must be present throughout the entire SC (2).

##### 4.1.2.3. Visibility

The capacity to comprehensively monitor the entire SC (2). The implementation of effective risk mitigation strategies depends on enhanced Visibility (VSB) throughout the chain (32). Additionally, another critical factor contributing to VSB within SCs is the ability to monitor signs of market changes.

##### 4.1.2.4. Redundancy 

This refers to strategically utilizing inventory and capacity so they can be used in case of an interruption (2). To increase resilience, it is important to enhance SC redundancy (33). This includes having multiple suppliers, the ability to switch between them, maintaining safety stock, and controlling market fluctuations through excess inventory.

##### 4.1.2.5. Market Position

This refers to a company’s or its products' standing in particular markets (8). One subfactor is customer loyalty, which extends beyond merely gauging future purchase intentions. Customer loyalty acts as a buffer against lost sales during disruptions and aids in recovering lost revenue. Various surveys across industries consistently show that retaining an existing customer is financially more advantageous, typically costing five to seven times less than acquiring a new one (34).

##### 4.1.2.6. Adaptability

The ability to re-route or adjust when faced with new opportunities or challenges. Companies that are adaptable can better respond to and manage changes, taking appropriate actions when necessary (35).

##### 4.1.2.7. Financial Strength

The capability to manage fluctuations in income and expenditures (8). For market advancement, maintaining a strong cash flow through FIN reserves is essential (36). Despite uncertainty, this improves SC performance (37).

##### 4.1.2.8. Collaboration

Collaboration is a key component of SC capabilities for resilience and is defined as the ability to work effectively with others for mutual benefit (37). 

Governments have also encouraged private businesses across various industries to modify their production methods to produce medical and respiratory equipment in response to the COVID-19 pandemic. Additionally, UNICEF collaborates with governments and the private sector to ensure developing countries have equitable and innovative access to COVID-19 diagnostic tools, treatments, and vaccines (38).

### 4.2. Quantitative Phase 

The Thurstone Scale included seven points, where '1' represented the lowest value and '7' the highest value, for the respondents' answers. Consequently, if the median score was above 4.0, it was considered a positive score, indicating valuable opinions. Table 1 summarizes the statistics for vulnerabilities and capabilities. Mean, median, mode, and standard deviations were calculated by averaging the respective constructs.

**Table 1.** Descriptive Statistics of Research Constructs

Main Constructs/Constructs	Numbers		Mean	Median	Mode	Standard Deviation
	Valid	Missing				
**Vulnerabilities**						
Total	547	0	4.97	5.12	5.12	0.88
ORG	547	0	4.30	4.50	5.00	1.10
IT	547	0	6.30	6.00	6.00	2.34
PRO	547	0	5.00	5.33	5.50	1.10
FIN	547	0	5.70	5.75	7.00	1.08
C/S	547	0	5.13	5.50	5.75	1.07
S/C	547	0	4.75	5.00	5.00	1.03
E/E	547	0	4.82	5.00	5.50	1.06
**Capabilities**						
Total	547	0	5.14	5.39	4.95	0.99
FLX	547	0	4.79	5.00	5.40	1.07
AGL	547	0	5.27	5.33	6.00	1.26
VSB	547	0	5.04	5.25	5.50	1.17
RDN	547	0	5.08	5.25	6.50	1.23
MP	547	0	5.23	5.33	6.00	1.26
ADP	547	0	5.32	5.50	5.00	1.10
FS	547	0	5.54	6.00	6.00	1.17
COL	547	0	4.89	5.00	4.50	1.05

Abbreviations: ORG, organizational; IT, information technology; PRO, procedural; FIN, financial; C/S, control-supportive; S/C, supplier-customer; E/E, external-environmental; FLX, flexibility; AGL, agility; VSB, visibility; RDN, redundancy; MP, market position; ADP, adaptability; FS, financial strength; COL, collaboration.

According to the Kolmogorov-Smirnov test, the data showed a non-normal distribution (all constructs: P = 0.000) (Appendix 4 in Supplementary File); therefore, using the median to analyze the data is preferable. Based on the questionnaire, all constructs had a median score above 4 in both the vulnerabilities and capabilities sections. Specifically, for vulnerabilities, information technology (IT) (6.00) scored the highest, followed by FIN (5.75), control/supportive (C/S) (5.50), procedural (PRO) (5.33), supplier/customer (S/C) (5.00), environmental/external (E/E) (5.00), and ORG (4.50). In terms of capabilities, FS (6.00) was rated the highest, followed by adaptability (ADP) (5.50), AGL (5.33), market position (MP) (5.33), VSB (5.25), and redundancy (RDN) (5.25). The lowest scores were for FLX (5.00) and collaboration (COL) (5.00).

As shown in Table 1, a general examination of vulnerabilities and capabilities reveals that respondents placed a higher value on capabilities (5.39) than on vulnerabilities (5.12).

The results suggest that individuals involved in pharmaceutical manufacturing firms in Iran place a high value on FIN vulnerability, second only to IT vulnerability. In contrast, FS was the most highly valued capability.

As shown in Table 2, the non-parametric Kruskal-Wallis test was used to determine whether there were significant differences in vulnerabilities and capabilities across different occupational sectors.

**Table 2.** Kruskal-Wallis Test Results ^a^

Main Constructs/Constructs	Test Statistics		Asymp. Sig.
	Kruskal-Wallis H	df	
**Vulnerabilities**			
ORG	11.33	5	0.05 ^b^
IT	17.32	5	0.00 ^b^
PRO	12.28	5	0.03 ^b^
FIN	10.52	5	0.06
C/S	2.30	5	0.81
S/C	12.69	5	0.03 ^b^
E/E	11.28	5	0.05 ^b^
**Capabilities**			
FLX	6.61	5	0.25
AGL	9.84	5	0.08
VSB	15.22	5	0.01 ^b^
RDN	19.47	5	0.00 ^b^
MP	22.55	5	0.00 ^b^
ADP	29.95	5	0.00 ^b^
FS	30.95	5	0.00 ^b^
COL	8.91	5	0.11

Abbreviations: ORG, organizational; IT, information technology; PRO, procedural; FIN, financial; C/S, control-supportive; S/C, supplier-customer; E/E, external-environmental; FLX, flexibility; AGL, agility; VSB, visibility; RDN, redundancy; MP, market position; ADP, adaptability; FS, financial strength; COL, collaboration.

^a^ Grouping variable: Occupation.

^b^ P ≤ 0.05.

The results for Organizational (ORG), IT, PRO, S/C, E/E, AGL, VSB, RDN, MP, ADP, and FS were considered significant (P < 0.05), indicating that these constructs varied across occupational domains. 

As shown in Table 3, the Mann-Whitney test was used to compare each pair of occupations across the ten constructs (Appendix 5 in Supplementary File). The results indicated that within the organization construct, the responses for three job pairs—commerce-marketing, marketing-production, and QC-technical/engineering—were significantly different. The commerce-production job pair exhibited differences concerning the technology construct. Additionally, significant differences were found in the external/environmental construct for the commerce-finance and production-QC job pairs, the VSB construct for the QC-finance job pair, the ADP construct for the marketing-production job pair, and the FS construct for the commerce-finance job pair.

**Table 3.** Mann-Whitney Test Results

Constructs Occupations	ORG	IT	PRO	S/C	E/E	VSB	RDN	MP	ADP	FS
**Commerce marketing**	0.07	0.05	0.01	0.02	0.00	0.01	0.00	0.01	0.01	0.00
**Commerce finance**	0.00	0.04	0.00	0.04	0.07	0.01	0.01	0.00	0.01	0.06
**Commerce technical/engineering**	0.02	0.02	0.03	0.05	0.00	0.03	0.00	0.00	0.00	0.00
**Commerce QC**	0.00	0.00	0.03	0.00	0.02	0.01	0.01	0.00	0.00	0.02
**Commerce production**	0.05	0.07	0.00	0.03	0.02	0.05	0.01	0.00	0.00	0.01
**Marketing production**	0.06	0.05	0.03	0.05	0.00	0.00	0.04	0.04	0.06	0.03
**Finance production**	0.00	0.04	0.02	0.01	0.02	0.02	0.04	0.04	0.01	0.02
**Production technical/engineering**	0.01	0.03	0.00	0.02	0.03	0.02	0.03	0.01	0.04	0.00
**Production QC**	0.01	0.00	0.00	0.00	0.06	0.02	0.00	0.04	0.00	0.00
**Marketing QC**	0.01	0.00	0.02	0.00	0.00	0.00	0.00	0.05	0.00	0.00
**QC finance**	0.01	0.00	0.02	0.01	0.02	0.06	0.00	0.02	0.00	0.01
**QC technical/engineering**	0.06	0.00	0.05	0.00	0.04	0.00	0.00	0.00	0.00	0.00
**Technical/engineering marketing**	0.01	0.02	0.01	0.01	0.01	0.02	0.04	0.01	0.04	0.01
**Technical/engineering finance**	0.01	0.05	0.01	0.05	0.00	0.00	0.03	0.03	0.00	0.00
**Finance marketing**	0.00	0.03	0.05	0.01	0.00	0.00	0.04	0.02	0.02	0.01

Abbreviations: ORG, organizational; IT, information technology; PRO, procedural; S/C, supplier-customer; E/E, external-environmental; VSB, visibility; RDN, redundancy; MP, market position; ADP, adaptability; FS, financial strength.

## 5. Discussion 

The study's results revealed vulnerabilities and issues within the PSC during the COVID-19 pandemic and proposed strategies to strengthen PSC resilience, particularly for manufacturers. These vulnerabilities affected various aspects of the PSC, contributing to a comprehensive understanding of the challenges faced in this field. The identification of these deficiencies was used to enhance the resilience of the PSC in the pharmaceutical sector.

The sub-themes and codes emphasized in the interviews also achieved high scores in the quantitative phase. This alignment between the qualitative and quantitative phases demonstrates the robustness and accuracy of this study.

The results from Table 1 showed a higher value for capabilities (5.39) compared to vulnerabilities (5.12), indicating that the SCR in pharmaceutical manufacturing companies was deemed acceptable. Financial strength, ADP, AGL, and MP played a significant role in the high score for capabilities.

Based on the results of the Kruskal-Wallis test, the 10 constructs yielded different outcomes across the occupational groups. Additionally, the Mann-Whitney test results in Table 3 revealed that seven pairs of occupational groups showed significant differences.

Information technology infrastructure plays a central role in ensuring the effectiveness of an organization’s information system. Organizations must identify and improve these areas and infrastructures to respond quickly to environmental opportunities and threats. Information technology provides several benefits to manufacturers, including enhancing SC agility, reducing cycle time, achieving higher efficiency, and ensuring timely product delivery to customers (39). Information technology was identified as a key vulnerability (6.00) in the study, but it also played a critical role in capability sub-constructs such as the speed of information exchange (AGL) (6.00), the ability to monitor and track materials (VSB) (6.00), staying informed about market changes (VSB) (6.00), and managing customer relationships (MP) (6.00). These sub-constructs achieved high scores, underscoring the significant impact and relevance of IT on them.

Sanctions as a sub-construct (6.00), whether during or before the COVID-19 crisis, emerged as a key factor affecting the vulnerability of the PSC and other industries. 

In macroeconomic crises or unstable conditions, political-economic sanctions on certain underdeveloped or developing countries can significantly weaken the PSC, making it more susceptible to internal and external disruptions (40). These adverse effects are amplified within the health system, directly impacting people’s access to essential medicines and medical devices (41). Despite claims of exemptions for medicines and medical equipment from politico-economic sanctions, patients' access to necessary medications and medical supplies is severely hindered by transport barriers, difficulties in cash transfers, and restrictions on investment (42).

Similar to this research, Kokabisaghi highlighted that political and economic sanctions have severely affected macroeconomic conditions, leading to a decrease in the value of the country's currency, reduced export earnings, restrictions on the import of raw materials needed by the industry, increased inflation, industry bankruptcies, and unemployment. This, in turn, results in greater vulnerability in the resilience of the PSC (43). Ghiasi et al. found that the embargo affected access to asthma treatments, reducing access to imports by 19% and locally manufactured medications by 42%. This finding aligns with the experts’ views in this study (44). Moret emphasized the harmful impact of sanctions on Syrian and Iranian citizens' ability to obtain and use medications. The qualitative stage of this research focused on the specific impact of sanctions and FIN instability, highlighting the negative effect of economic sanctions on the country’s business cycle (45).

This study found that FIN concerns scored the second highest in the vulnerabilities section (5.75) and the highest in the capabilities section (6.00). These FIN vulnerabilities are comparable to Jaberidoost et al.'s study, which identified FIN risk as the highest risk (46).

In the common sub-constructs between our study and the pre-COVID-19 study on the resilience of the PSC in Malaysia (47), E/E, FLX, and COL scores on the questionnaire were acceptable. However, the VSB and ADP scores were not. Initially, we used expert interviews to gather strategies for addressing the COVID-19 situation, and based on these interviews, we applied a quantitative methodology. In contrast, the Malaysian study used only a quantitative approach.

In contrast to our study, Pettit et al.'s research (48) found that in seven instances of vulnerabilities, the external/environmental category received the highest ranking (rank 1), while the customer/supplier category was ranked 6th. In the capabilities section, MP ranked first out of fourteen, followed by FS at rank 3, VSB at rank 9, FLX at rank 10, ADP at rank 11, redundancy at rank 13, and COL at rank 14. The current COVID-19 crisis, which highlighted the significance of SCR through challenges faced in Iran and globally, was the primary motivation for our research. We decided to study the PSC's resilience during the COVID crisis by consulting the nation’s PSC experts and following up with a questionnaire. This research was inspired by Pettit’s study on SC resilience across various industries.

In the interviews, non-expert decision-making was frequently mentioned, with many interviewees emphasizing that this made the SC more vulnerable during the COVID crisis. This sub-construct received a score of 6.00 in the questionnaire. Non-expert decisions were driven by the lack of attention to expert opinions, such as the delayed declaration of the epidemic in the country and the establishment of a very high number of vaccine production platforms without proper feasibility studies, pharmacoepidemiological analyses, or other necessary studies.

### 5.1. Limitations and Future Research Directions 

The limitation of our study lies in the unique circumstances of the COVID-19 pandemic and its focus on a specific part of the SC (pharmaceutical manufacturing companies). Since the study was conducted after the pandemic had subsided, information bias was inevitable.

Future research could explore the applicability of the identified resilience strategies in other industries. Additionally, longitudinal studies could assess the long-term effectiveness of the resilience strategies implemented by pharmaceutical companies. Overall, this study serves as a foundation for further research on SCR in the pharmaceutical sector and underscores the importance of proactive resilience strategies in mitigating the impact of future disruptions.

### 5.2. Novelty of This Study 

This study presents several key aspects of novelty:

#### 5.2.1. Contextual Focus on the Iranian Pharmaceutical Supply Chain 

This study specifically examines the resilience of the PSC within the context of Iran. While existing literature on SCR, particularly in the pharmaceutical sector, is available, the focus on Iran provides unique insights into the challenges and strategies for resilience within a specific geopolitical and economic environment. This contextual specificity enhances the relevance and applicability of the findings for stakeholders in Iran's pharmaceutical industry.

#### 5.2.2. Mixed-Methods Approach 

By utilizing both qualitative insights from expert interviews and quantitative data from surveys, this comprehensive approach allows for a thorough investigation of resilience variables within the PSC. With findings drawn from multiple sources, the study provides valuable insights into resilience dynamics, enriching the academic discourse on SCR.

Overall, the novelty of this study lies in its contextual focus, mixed-methods approach, comprehensive analysis of vulnerabilities and capabilities, and sector-specific insights, all of which contribute to advancing the understanding of SCR within the Iranian pharmaceutical industry.

### 5.3. Conclusions

The increasing complexity and uncertainty in the business environment have pushed disaster mitigation strategies to their limits, highlighting the importance of SCR as a key driver of future business success. The COVID-19 pandemic has undoubtedly contributed to the heightened attention and accelerated progress in this field. Resilience is not a one-time solution but a continuous process.

This research emphasizes the significance of an organization’s financial characteristics, both in terms of weaknesses and strengths. Strengthening these areas and permanently speeding up information flow through rapid response mechanisms will enable the PSC, especially during crises like pandemics and sanctions, to become more resilient. The study primarily focused on manufacturers as the central part of the PSC. It is recommended that similar studies be conducted in other segments of the PSC to gain a more comprehensive understanding.

ijpr-23-1-152723-s001.pdf

## Data Availability

The dataset presented in the study is available upon request from the corresponding author during submission or after publication.
