# Supplementary material for: The COVID-19 Pandemic and the Resilience of the Pharmaceutical Supply Chain: Lessons from Past Experiences and Strategies for the Future
Source: Iran J Pharm Res. 2024 Nov 5;23(1):e152723. doi: 10.5812/ijpr-152723 (PMC11742386; doi:10.5812/ijpr-152723)
Supplement: ijpr-23-1-152723-s001.pdf [file ijpr-23-1-152723-s001.pdf]

Appendix 1

Respondents' Demographic Data

Variables		No. (%)
**Gender**		
	Male	304 (55.6)
	Female	165 (30.2)
	Missing	78 (14.3)
**Age (year)**		
	Less than 35	49 (9)
	36 - 40	157 (28.7)
	41 - 45	157 (28.7)
	46 -50	79 (14.4)
	51 -55	14 (2.6)
	Missing	91 (16.6)
**Education**		
	Postgraduate Diploma	13 (2.4)
	Bachelor	136 (24.9)
	Master	226 (41.3)
	Doctorate	96 (17.6)
	Missing	76 (13.9)
**Occupation**		
	Commerce	74 (13.5)
	Marketing	39 (7.1)

Finance	83 (15.2)
Technical/Engineering	82 (15.0)
QC	65 (11.9)
Production	83 (15.2)
Technical manager	19 (3.5)
QA	21 (3.8)
Missing	81 (14.8)

Appendix 2

Reliability Statistics

Main Constructs	Constructs	Cronbach's Alpha	Number of Subconstructs
Vulnerabilities	ORG	0.752	6
	TCH	0.748	2
	PRO	0.884	6
	FIN	0.865	4
	C/S	0.810	4
	S/C	0.774	4
	E/E	0.793	6
Capabilities	FLX	0.839	5
	AGL	0.877	3
	VSB	0.829	4
	RDN	0.894	4

MP	0.856	3
ADP	0.881	4
FS	0.890	3
COL	0.844	6

**Appendix 3**
**Some expert sentences about the related sub-theme**

Vulnerabilities:

1. Organizational

"The information was not comprehensive and centralized anywhere, for example, the statistics of patients and deaths were in one site, the treatment protocol and the drugs that should be prescribed were in another place, and the information was scattered, but in the international protocol, these were in one place". {P_6}

2. Information Technological

"During the COVID-19 pandemic, the technology infrastructure within the department was inadequate. This inadequacy led to outdated information and poor information-sharing practices. It's a widespread problem across different sectors, including both the overarching government level and smaller micro-level sectors, including businesses". {P_3}

"The inadequate and uneven coverage of internet services across the country has resulted in obstacles to the seamless transfer of information." {P_9}

3. Procedural

“In the beginning of the Corona crisis, we faced a serious problem in providing medical supplies such as masks, gloves and clothes”. {P_{12}}

4.<u>Financial</u>

“According to people's attention to COVID-19 drugs, several companies that were not involved in those drugs production or distribution faced cash shortages, which amplified the impact of the crisis. Distributors gave priority to partnerships with companies associated with COVID-19 drugs, leaving companies producing non-coronavirus drugs to meet lack of liquidity.” {P5}

5. <u>Control / Supportive</u>

“During the peak of the Covid outbreak, there was a weakening of control over product quality, which led to substandard items such as masks, PPE, and disinfectants entering the market" {P_3}.

"Infrequent and insufficient meetings involving supply chain professionals resulted in a lack of science-based expert decisions”. {P_{15}}

6. <u>Supplier / Customer</u>

“In the COVID-19 crisis, the focus was on effective drugs for the prevention and treatment of COVID-19. As a result, challenges arose in drug supply for specific diseases, especially chronic diseases such as diabetes, which require lifelong medication. The scarcity of insulin became a critical issue, worrying those who depend on it”. {P_{16}}

7. <u>Environmental / External</u>

“Medicines purchase has been made a very complex process which is now extremely costly as a result of embargo banking. Increased sanctions undoubtedly add to the extra costs, aggravate the supply operational links, and consequently result in longer lead times”. {P_{11}}

“One of the other important side effects of sanctions is related to the Letter of Credit transactions across banking communication delays between Iran and other countries”. {P_{18}}

Capabilities:

1. <u>Flexibility</u>

“We did not have significant financial resources to deal with the Covid crisis, so we strategically decided on special drugs for the treatment of corona patients (antiviral, anticoagulant, corticosteroids, etc.), and also to plan the necessary consumables, we had about 47 medicines and 53 consumables that were prepared and entered in the hospital HIM and the related issues were solved to a large extent”. {P_{20}}

2. Agility

"The increase in the speed of production and preparation of vaccines led to a wide coverage of vaccination in the country". {P_{10}}

3. Visibility

"During the Covid-19 crisis, our company evaluated the health of each sector within the supply chain and the status of the goods with the Business Intelligence (BI) and Machine Learning unit. We were able to actively monitor these aspects". {P_{19}}

4. Redundancy

"It was a good achievement to get help from the faculty of pharmacy and human resources of pharmacy students to produce alcohol and disinfectants". {P_9}

5. Market position

"Patients/customers often recognize the specific therapeutic efficacy associated with a particular brand of medicine and claim that other brands do not have the same effect and therefore refuse to use them" {P_{17}}

6. Adaptability

"The experience of border closures, such as the situation in China, and instances of non-compliance in the supply of raw materials, similar to what happened in India, prompted us to stockpile at least one month's worth of medicines for upcoming shipments. This proactive approach ensures that if these issues were to recur, we would be well prepared and avoid any disruption to the supply chain" {P_{14}}.

7. Financial strength

"Thanks to our sufficient financial reserves, we could during the Covid-19 crisis meet the demand for essential goods without facing any significant challenges". {P_1}

8. Collaboration

"During the management of COVID-19 cases, the sharing of critical information played a crucial role in preventing the recurrence of events and complications in different locations". {P_2}

Appendix 4

Test of normality

Constructs	Kolmogorov-Smirnov[a] Statistic	df	Sig.
ORG	0.109	547	0.000
TECH	0.080	547	0.000
PRO	0.134	547	0.000
FIN	0.138	547	0.000
C/S	0.141	547	0.000
S/C	0.135	547	0.000
E/E	0.109	547	0.000
FLX	0.105	547	0.000
AGL	0.127	547	0.000
VSB	0.132	547	0.000
RDN	0.114	547	0.000
MP	0.135	547	0.000
ADP	0.109	547	0.000
FS	0.187	547	0.000
COL	0.109	547	0.000

Appendix 5

Mann-Whitney test

Table 3: Mann-Whitney test

Occupations \ Constructs	Mean Ranks ORG	TCH	PRO	S/C	E/E	VSB	RDN	MP	ADP	FS
**Commerce**	**57.02**	57.76	60.45	59.30	62.93	60.92	61.62	60.19	60.49	61.43
**Marketing**	**56.96**	55.56	50.45	52.63	45.76	49.56	48.23	50.95	50.38	48.60
**Commerce**	70.59	76.82	84.76	76.74	**78.92**	73.72	85.22	93.07	83.93	**79.61**
**Finance**	85.50	80.94	73.87	81.01	**79.07**	83.71	73.46	66.46	74.61	**78.46**
**Commerce**	74.63	75.08	80.83	77.05	85.83	81.43	86.80	94.30	88.41	92.89
**Technical/Engineering**	81.99	81.59	76.40	79.80	71.88	75.86	71.01	64.24	69.56	65.51
**Commerce**	63.74	58.59	72.42	62.97	73.58	65.14	65.20	79.54	63.76	66.25
**QC**	77.12	82.98	67.25	78.01	65.92	75.54	75.47	59.14	77.11	74.27

**Commerce**	80.59	**79.04**	89.87	82.19	83.41	78.15	84.77	90.91	87.70	85.32
**Production**	77.58	**78.96**	69.31	76.16	75.07	79.76	73.86	68.38	71.24	73.36
**Marketing**	**61.97**	60.01	65.54	59.74	52.12	52.82	59.27	64.45	**61.76**	57.50
**Production**	**61.28**	62.20	59.60	62.33	65.91	65.58	62.55	60.11	**61.38**	63.38
**Finance**	92.42	85.54	87.75	88.63	87.69	87.92	85.60	81.27	89.41	87.67
**Production**	74.58	81.46	79.25	78.37	79.31	79.08	81.40	85.73	77.59	79.33
**Production**	77.92	79.81	72.18	78.64	85.78	87.21	85.46	88.17	84.56	93.00
**Technical/Engineering**	88.15	86.23	93.95	87.41	80.19	78.74	80.51	77.76	81.42	72.88
**Production**	69.45	63.99	67.86	65.61	**74.71**	71.05	65.07	73.08	61.46	65.88
**QC**	80.95	87.92	82.98	85.85	**74.23**	78.91	86.55	76.32	91.15	85.51
**Marketing**	46.46	39.91	48.26	41.01	45.45	40.01	40.91	53.63	39.08	41.69
**QC**	56.12	60.05	55.05	59.39	56.73	59.99	59.45	51.82	60.55	58.98
**QC**	69.07	85.25	78.36	80.28	70.19	**74.79**	86.45	79.09	65.52	70.18
**Finance**	78.75	66.08	71.48	69.98	77.87	**74.27**	65.14	70.90	65.52	70.18
**QC**	**74.65**	84.67	73.05	82.90	76.45	85.01	88.60	81.64	92.71	93.32
**Technical/Engineering**	**73.49**	65.54	74.76	66.95	72.06	65.27	62.43	67.95	59.17	58.69
**Technical/Engineering**	64.13	63.23	64.16	63.91	64.73	63.63	62.01	57.37	60.08	58.00
**Marketing**	54.42	56.32	54.35	54.88	53.15	55.47	58.88	68.63	62.94	67.31
**Technical/Engineering**	77.87	84.20	87.98	81.45	75.99	74.06	80.38	79.71	75.20	70.28
**Finance**	88.07	81.81	78.08	84.54	89.92	91.83	85.59	86.25	90.70	95.57
**Finance**	66.81	63.19	62.04	65.32	66.92	67.66	62.81	59.26	63.81	64.79
**Marketing**	50.21	57.90	60.36	53.37	49.96	48.38	58.71	66.27	56.59	54.50
